# The State of the Art of Radiotherapy for Non-melanoma Skin Cancer: A Review of the Literature

**DOI:** 10.3389/fmed.2022.913269

**Published:** 2022-06-27

**Authors:** Sofian Benkhaled, Dirk Van Gestel, Carolina Gomes da Silveira Cauduro, Samuel Palumbo, Veronique del Marmol, Antoine Desmet

**Affiliations:** ^1^Department of Radiation-Oncology, Institut Jules Bordet-Université Libre de Bruxelles, Brussels, Belgium; ^2^Department of Radiation-Oncology, Jolimont Hospital, La Louvière, Belgium; ^3^Department of Dermatology, Erasme Hospital, Université Libre de Bruxelles, Brussels, Belgium

**Keywords:** non-melanoma skin cancer, squamous cell carcinoma, basal cell carcinoma, radiotherapy, brachytherapy

## Abstract

Due to the general aging population and the fashion trend of sun exposure, non-melanoma skin cancer (NMSC) is rising. The management of NMSC is difficult and necessitates a multidisciplinary team (i.e., pathologists, dermatologists, medical oncologists, surgeons, and radiation oncologists). When surgery is not an option or will cause unacceptably functional morbidity, radiation therapy (RT) may be a preferable tissue-preserving option. Whether used alone or in conjunction with other treatments, RT has been shown to be quite effective in terms of cosmetic results and local control. Contact hypofractionated RT, brachytherapy, and electronic brachytherapy are all promising new treatments. However, rigorous, randomized trials are missing, explaining the disparity in dose, fractionation, and technique recommendations. Therefore, it is essential that interdisciplinary teams better understand RT modalities, benefits, and drawbacks. Our review will provide the role and indications for RT in patients with NMSC.

## Introduction

Non-melanoma skin cancers (NMSCs) are the most common malignant tumors diagnosed worldwide (1–3). Due to ultraviolet light (UV) exposure, over 95%of cancers are located in the head and neck (H&N) region (nose, ears, eyelids, and lips). Basal cell carcinoma (BCC) and squamous cell carcinoma (SCC) represent 99% of NMSC (2, 4). Their incidence rates increase each year (1, 5). It should be noted that half of these patients are above 65 and that half will develop a second primary NMSC within 5 years. Even though NMSCs are typically thought to have a fair prognosis and are curable, they represent a significant health care burden worldwide due to their increasing incidence (5, 6). Mohs micrographic surgery (or its variants) remains the most common practice with good cosmetic and oncological results. However, surgery for larger tumors can be mutilating and/or require complex reconstruction techniques and general anesthesia.

Radiation therapy (RT) has always played an essential role in the management of NMSC (2, 4). It can be used at any stage of the disease, with curative or palliative intent, as an exclusive, adjuvant, or concomitantly with systemic treatment (2, 7, 8). RT should only be avoided in rare genetic syndromes such as ataxia-telangiectasia, connective tissue disease (i.e., Verrucous carcinoma, Li-Fraumeni), and basal cell carcinoma/Gorlin syndrome (4, 9). Although Xeroderma pigmentosa raises concerns about developing additional NMSC, it is not an absolute contraindication to RT (9).

The only randomized study comparing surgery and exclusive RT was published 25 years ago by Avril et al. (10). This study on facial BCCs favored surgery both aesthetically and oncologically (10). However, the number of complex surgical procedures (reconstructions and re-excisions) in the surgical arm, as well as the heterogeneous, outdated techniques and doses in the RT arm, are significant biases. Therefore, the established role of RT (i.e., dose, fractionation, techniques) in NMSC is essentially based on observational cohort studies without concrete evidence.

Moreover, the present high-tech RT modalities are often misunderstood or not well known by the other members of the highly specialized multidisciplinary team (i.e., pathologists, dermatologists, medical oncologists, surgeons) involved in NMSC management. In the absence of a radiation-oncologist at the multidisciplinary tumor board, RT is likely underused. In this study, we will review and synthesize the modern RT indications and techniques recommended in the NMSC treatment.

## Materials and Methods

We performed PubMed, Medline, EMBASE, and Cochrane Library database searches using “non-melanoma skin cancer,” “NMSC,” “squamous cell carcinoma,” “SCC,” “basal cell carcinoma,” “BCC,” “cancer,” “radiotherapy,” “brachytherapy,” “high-dose-rate brachytherapy,” “HDR brachytherapy,” low-dose-rate brachytherapy,” or “LDR brachytherapy.” All relevant articles were reviewed and incorporated by three authors (AD, SB and CG) as appropriate.

## Classic External Beam Radiotherapy

This section will go over external beam irradiation with high-energy photons or electrons delivered by a linear accelerator as the sole treatment (exclusive), after surgery (adjuvant), and/or in combination with systemic treatment (concomitant).

## Exclusive External Radiotherapy

Although surgery is favored for resectable lesions (7, 8), external RT could provide excellent healing effects in early-stage NMSC (4). The most common indications for RT include older patients with comorbidities, incurable disease, and tumors involving a sensitive part of the face (nose, canthus, brows, and lips) that may result in a functional or cosmetic deficit following surgery (11). Many publications have confirmed the excellent results of exclusive RT. The review of Caccialanza et al. of 986 eyelid SCC and BCC treated with exclusive RT showed excellent outcomes with a 5-year local control (LC) of 96.4% (12). In addition, the Princess Margaret Hospital found in 334 ear tumors (SCC and BCC) treated exclusively by RT a 2-year LC rate of 87% with only 7% severe late toxicities (13) (Table 1).

**Table 1:** Classic external beam radiotherapy.

**Author**	**Year**	**Histology**	**Exclusive/** **Adjuvant**	**N. lesions** **N. patients**	**N. Fractions**	**Dose/Fraction**	**Total Dose**	**Local control** **2 years**	**Local control 5 years**	**5-year Disease-Free Survival**
Silva et al. (13)	2000	BCC (201) SCC (122) Basosquamous (11)	Exclusive	334 lesions 313 patients	1 5 10 20–30	17.5–20 Gy 7 Gy 4.2–4.5 Gy <3 Gy	17.5–20 Gy 35 Gy 42.5–45Gy 50–65 Gy	87%	79%	
Veness et al. (14)	2005	SCC with metastatic lymph nodes	Adjuvant	167 lesions 167 patients	25–37	2 Gy	50–74 Gy			73% vs. 54% (surgery alone).
Caccialanza et al. (12)	2013	BCC SCC	Exclusive	986 lesions 905 patients					92–96%	
Porceddu et al. (15)	2018	SCC (310)	Adjuvant Adjuvant +chemotherapy	157 patients 153 patients	30–33	2 Gy	60–66 Gy	88% 89%	83% 87%	67% 73%
Voruganti et al. (16)	2021	BCC (14) SCC (77) Others (15)	Exclusive	112 lesions 106 patients	4–6	5–8	25Gy 32–35 Gy 40 Gy 45 Gy 48–50 Gy	67%		
De Felice et al. (17)	2021	SCC (23)	Exclusive	23 lesions 18 patients	8	7–8	56–64 Gy			23.5% (2–year)

Several characteristics, including recurrent disease, bone infiltration, and peri-neural infiltration (PNI), are highly associated with decreased LC and disease-specific survival when only RT is used. In the case of lymph node involvement, surgery followed by RT should be used in most cases (see the indication in the adjuvant radiotherapy section). There is no high-quality published evidence of the efficacy of exclusive RT for curative treatment (7, 8). If the disease is inoperable, radiation may be recommended, but systemic therapeutic approaches and/or clinical trials must also be considered (2, 7, 8).

The radiation oncologist determines his prescription (dose and fractionation) based on various considerations, including the general patient's status, the location and volume of the lesion, the surrounding organs, and the available RT resources. The best RT dose is currently unknown due to a lack of prospective studies; nevertheless, many schemes exist. Accelerated or hypofractionated RT (AF/HFRT) is preferred for older or fragile patients (17–19). Furthermore, HFRT gained importance during the SARS-CoV-2 global pandemic (3). For NMSC up to 5 cm, HFRT can be delivered daily, on alternate days, or weekly, providing the same biological dose in fewer fractions (5–7 Gy/fraction). It provides a high LC (90–100%) (18, 20) while being well-tolerated, with only a few (if any) developing acute moist desquamation (18). A meta-analysis of 9.729 NMSC patients treated with HFRT showed “good” cosmetic results in 92% of patients after a median follow-up of 36 months (20). Telangiectasia and hyperpigmentation were the most reported, with no grade 4–5 RTOG toxicities (toxicity criteria of the radiation therapy oncology group) (20).

Current RT schemes may include the following:

- Tumor <2 cm: 30 Gy in 5 fractions over 2–3 weeks; 40 Gy in 2 weeks; 50–55 Gy or 3–4 weeks to 60-64 Gy over 6-7 weeks.
- Tumor ≥2 cm, T3-T4, infiltration of bone or deep tissue: 45–55 Gy in 3–4 weeks to 60–70 Gy over 6–7 weeks (7, 8).

In 2018, a systematic review of 12.337 NMSC treated mainly with HFRT (93% of patients) was performed (18). They found a significant heterogeneity in patients' selection (mean age: 72 years); tumor location (head and neck: 96%); lesion size (1–5 cm); histology (BCC: 75%); RT setting (definitive: 97%); and RT modalities (external, brachytherapy, or both). Toxicities were not well documented. The weighted-mean total dose calculated was 38 Gy, with 7.95 Gy per fraction over 2.98 weeks. The authors concluded that HFRT with 5–7 Gy per fraction up to 30–40 Gy results in high local control and tolerable toxicity. The prescription might be determined on a case-by-case basis and based on the practitioner's experience.

## Adjuvant Radiotherapy

Although therapies such as topical drugs, surgery, or RT are curative for the vast majority of BCC patients, they are rarely treated postoperatively (21). Patients with positive margins should be discussed in a multidisciplinary team and surgical excision with tumor-free histological margins should be performed (22–24). Radiotherapy is based on patient condition, recurrence, or perineural infiltration (PNI) disease (21). Adjuvant RT should be considered for persistently positive or close margins after multiple resections, for T4 with extensive infiltration in bone or soft tissues, in the case of lymph node metastasis (which is extremely rare) or PNI (extensive or clinical) (7, 8). Adjuvant RT should start ideally within 6 weeks of surgery, as an extrapolation from head and neck (H&N) mucosal SCC (4), but the delay is often longer due to the postoperative and sometimes the post-reconstruction status of the frail patient.

SCC, on the other hand, may have a high rate of local recurrence following surgery alone, with a risk of metastasis of 3.7% and a risk of disease-specific mortality of 2.1%. As shown by the NCCN guidelines, the value of adjuvant radiation in the setting of clean surgical margins is widely debated (8). The lack of precise recommendations for adjuvant RT leads to variation among physicians and institutions. It is important to note that the majority of studies defining risk factors for local recurrence of NMSC are restricted to the H&N area (4). Adjuvant RT should be considered in the following cases: T4, positive margins, clinical PNI, or patients with two or more intermediate risk factors (4, 8, 25). Intermediate risk factors are tumor > 2 cm, poorly differentiated, > 4 mm depth, subcutaneous fat infiltration, desmoplastic growth characteristic, recurrent tumor, ear or upper lip localization, microscopic PNI, lymphovascular infiltration (LVI), immunosuppressed status (IS) (4, 8, 25). A multi-institutional retrospective study (2008–2016) of 349 patients with advanced H&N SCC assessed the benefits of adjuvant RT (25). In a multivariate analysis, adjuvant RT (191 patients) was associated with improved overall survival (OS) (Hazard ratio [HR] 0.59; 95% CI, 0.38–0.90) in the entire cohort. Patients with PNI (39%) and regional disease (37%) benefitted the most from adjuvant RT. This is the largest series examining (in a direct comparison with surgery alone) the benefit of adjuvant RT. However, those results have to be interpreted with caution due to the retrospective nature, the number of recurrent diseases (58.5%), the absence of a clear cut off to define high-risk patients, and the absence of RT information (dose, field, techniques, etc.) and indication (“RT was delivered at the discretion of the radiation oncologists”) (25). A meta-analysis did not find significant differences in 3.867 (poor outcomes with clear margins) cSCC patients between surgery alone and surgery plus adjuvant RT (26). The effect of adjuvant RT in these high-risk cases of cSCCs remains unclear, and randomized studies are needed.

In SCC, the incidence of lymph node (LN) metastasis is around 1–4%, with two primary regions at highest risk, the ear (HR 1.70; 95% CI, 1.42–2.03) and lip (HR 1.85; 95% CI, 1.29–2.63) (27). High-risk patients are mostly defined by the recurrence setting, IS, unfavorable location, tumor size ≥2 cm, PNI, and poorly differentiated tumor. In operable node-positive patients, LN dissection followed by postoperative RT is the standard of care (8). However, when the LN are involved, the locoregional recurrence rate (LRR) ranges between 30–50%, and cancer mortality rises to 30% (8, 19). These suboptimal outcomes highlight the need for a multimodal approach, including surgery and adjuvant RT for advanced NMSC. A review of 167 SCC patients with positive lymph nodes (cervical and/or intraparotid) showed a significantly decreased LRR (20 vs. 43%; p not mentioned) and increased 5-year disease-free (73 vs. 54%; *p* = 0.004) and OS (50 vs. 58%; p not mentioned = 0.003) with adjuvant RT compared to surgery alone (14) (Table 1).

Similar to H&N cancer, adjuvant RT to the LNs is not necessary if the infiltration concerns only one LN, <3 cm, without extracapsular extension (ECE) after neck surgery in an immunocompetent patient, the LRR being 5% (28). Current prognostic classification systems (even the revised Brigham and Women's Hospital classification) do not take into account the patient's IS. Immunosuppression is, in fact, a significant risk factor for local recurrence (29). Thus, postoperative RT should be strongly rigorously for this population, especially in early-stage diseases. The dose and fractionation for the adjuvant treatment of the primary tumor vary from 60–64 Gy over 6–7 weeks to 50 Gy over 4 weeks. For the lymph node areas, it varies from 50–60 Gy over 5–6 weeks (after LN dissection and negative margins without ECE) to 60–66 Gy over 6–7 weeks (in the case of positive margins or ECE) (4, 8) In the case of substantial PNI (detectable on imaging), definitive doses should be applied, ideally with conventional fractionation (4). The first consensus contouring recommendations in the postoperative management of H&N SCC were recently published by the H&N Cancer International Group (HNCIG) (30).

## Exclusive/Adjuvant Chemoradiotherapy

There is no convincing evidence of the use of concurrent chemotherapy in both adjuvant and exclusive chemoradiotherapy for high-risk SCCs, with the literature consisting mainly of isolated case reports (31, 32). Without randomized trials on a concomitant treatment for high-risk SCC, the proposal is based on H&N studies, where the adjuvant benefit is limited to positive margins and ECE (33–35). In 2018, the TROG 0501 trial randomized 321 patients between concurrent adjuvant chemoradiotherapy (CCRT) *vs*. adjuvant RT alone in patients with high-risk SCCs (15). RT was used 6 weeks after surgery (60–66 Gy; 30–33 fractions) using three-dimensional conformal RT and intensity-modulated radiation therapy (IMRT). Chemotherapy consisted of weekly (*n* = 6) carboplatin (AUC2), not cisplatin. With a median follow-up of 60 months, there was no benefit of CCRT over RT. There was also no statistically significant difference in freedom from locoregional recurrence (HR 0.84; 95% CI, 0.46–1.55), DFS, or OS.

A small number of BCC patients will develop advanced diseases for which treatment can be challenging (21, 36). However, metastatic disease is scarce (<0.6%) and arises more frequently with large, aggressive, untreated, recurrent BCCs (21).

The hedgehog pathway inhibitors (*Vismodegib and Sonidegib)* are the first Food and Drug Administration (FDA) and European Medicines Agency (EMA) approved systemic treatment for advanced stages of BCC (2). The long-term update of the ERIVANCE BCC trial showed 39 monthly response rates between 48.5 and 60.3%, with good median response durations (14.8–26.2 months) (37). However, toxicities (grade ≥ 3: 55.8%) prevent further treatment in some patients. These results have been confirmed in the STEVIE trial (2). Hedgehog pathway inhibitors, are frequently associated with resistance and considerable toxicity, making long-term use of these drugs challenging (36). Future practices could be the combination of RT with Vismodegib for very high-risk BCC (2, 38). In 2015, a case report (*n* = 2) showed that the combination of Vismodegib with external radiotherapy (photons or electrons) in the H&N region was achievable without significant side effects (39).

The majority of SCC and BCC patients have hypermutated tumors due to persistent skin damage from UV, and the disease risk is enhanced in IS individuals (40). SCC has a high tumor mutation burden, which may be 5–15 times higher than in non-cutaneous malignancies, and they are more likely to respond to immunotherapy (40).

*Checkpoint inhibitors (ICI)* show promising results in metastatic cutaneous SCC and BCC (36, 41). Recently, the FDA and EMA have authorized Libtayo (Cemiplimab, PD-1 inhibitors) for metastatic or locally advanced SCCs in patients who are not candidates for surgery or RT, according to Study 1540 (phase I–II) (41). In addition, they also authorized Cemiplimab for patients with locally advanced or metastatic BCC. The latter have progressed or are intolerant to a hedgehog pathway inhibitor, according to Study 1620 (phase II) (36).

In both trials, patients had a median age of 70–73 years, and the majority were men with primary tumors of the H&N region (64–89%) (36, 41). The majority of these patients had a partial (25–50%) or stable response (15–49%). Curiously, the median response time for SCC was less than for BCC (1.9 vs. 4.3 months). There is an emerging paradigm that immunotherapy does have the best treatment effectiveness when administered earlier. Nonetheless, the mechanisms associated with response in SCCs and BCCs are unknown, and more investigations are needed. Combining RT with immunotherapy could shift the balance in favor of the immune response by activating and inhibiting multiple pathways (42, 43). In the 1540–1620 studies, 50–77% received prior RT, but no details were provided (i.e., techniques, prescription, or delineation) (36, 41).

In fact, RT, such as Stereotaxic Body Radiotherapy (SBRT), could effectively target the metastasis/resistance/progression, especially when the patient discontinued systemic treatment due to an adverse event or developed a repeated/induced oligo- recurrence/ progression/persistence disease. Although oligometastasis in SCC has yet to be documented, it is a reasonable possibility.

A systematic review found that the ICI + SBRT combination resulted in an LC of 75–91% and a 1–45% distant/abscopal response with 0–34% grade 3 toxicity in patients with extracerebral metastases from different histologies (including melanoma) (44). The heterogeneity (i.e., histology, population size, number and lesion location, prescription, systemic treatment) makes it challenging to draw any firm conclusions (44). However, some clinical conditions that make RT difficult are known to radiation oncologists, such as re-irradiation, poor patient compliance, and connective tissue disease. Nonetheless, there is a high likelihood that RT will be delivered effectively in more cases, and it would be appropriate to administer ICI following SBRT.

Because ICI in NMCS causes enthusiasm, closer monitoring should be considered for those patients. As a result, in future research, the numbers and sites of irradiated tumors may be used as stratification criteria to assess the potential synergism further. Similarly, according to two-phase II (Keynote 629-Carskin), Pembrolizumab (200 mg every three weeks) is now the second FDA-EMA PD-1 inhibitor approved for the treatment of advanced SCC (45, 46). Future studies may clarify the role of immunotherapy in the treatment of primary NMSCs(2, 41, 47, 48).

Regarding other drugs, there is also no strong, available data assessing the role of *EGFR inhibitors* administered with or without RT for cutaneous SCCs (2, 49). Overall, EGFR inhibitors have a modest effect as monotherapy or in combination, with an overall response rate between 7–50% and a median PFS of around 4–25 months (2, 50). Avelumab, either alone or in combination with Cetuximab, is being investigated in advanced SCC (NCT03944941). However, the potential RT-ICI synergism might be challenging to identified. Indeed, 2 weeks or 6 months prior to the previous RT-CCRT treatments are required in this phase II.

## Palliative-Symptomatic Radiotherapy

The World Health Organization defines palliative care as “enhancing patient quality of life and decreasing clinical discomfort.” Palliative RT is typically used when definitive therapy is contraindicated or after curative treatment, depending on the patient and/or tumor feature recurrence (51, 52). Palliative RT has the potential to decrease cancer-related symptoms such as pain, bleeding, ulceration, and neurological symptoms. To minimize toxicity while keeping a high dose to the target, we used fractionated approaches; typical schemes include 7–10 Gy in 3–10 fractions, with an LC of roughly 90% at 2–5 years (52). Despite the multiple treatment regimens, telangiectasia, pigmentation, fibrosis, and treatment-field ulceration were observed in fewer than 10% of NMSC patients (52). Barnes et al. reported a 48% local and 61% symptomatic control after a median follow-up of 17 weeks (51). They used 24 Gy in three fractions (biological equivalent dose, BED_10_: 43.2 Gy) once a week in 28 elderly NMSC patients. The most prevalent symptoms were bleeding and discharge, and the majority of the tumors were found on the head (cheek, forehead/temple, ear) with a 2–5 cm lesion in 52% cases. Voruganti et al. found 1–2 year LC rates of 78 and 67%, respectively, in 106 unfit patients with locally advanced H&N skin cancer using SBRT (25–50 Gy, 4–6 fractions) (16). Patients with aggressive, bulky tumors ± nodal involvement could benefit from a higher biological dose (40–50 Gy, BED_10_: 72–100 Gy) to obtain better LC. According to De Felice et al., weekly HFRT (56–64 Gy in 7–8 fractions) is safe and cost-efficient in elderly unfit SCC patients (17). Complete and partial response rates at 12 weeks were 65 and 30%, respectively (17). No severe toxicity was reported. Palliative RT involves a rigorous quality of life assessment, including post-treatment cosmesis and psychosocial stress, which must be balanced against treatment outcomes (52). In general, the use of repeat RT in the treatment of NMSC is not reported, and reports in the literature are scant.

## Radiotherapy and Surgery

The interaction between preoperative, exclusive, or adjuvant RT and surgery has long been controversial (53, 54).

A meta-analysis published in 2022 found that preoperative RT was related to a higher risk of total (OR 1.68; 95% CI, 1.41–2) and partial (OR 1.75; 95% CI, 1.39–2.2) free flap failure (53). However, most of the studies were retrospective, including a total of 6.332/18.776 irradiated flaps from 17.532 patients, focusing on the breast and the H&N region. Furthermore, H&N patients were more likely (than breast patients) to experience complete flap failure. Nevertheless, no dosimetric outcomes, time intervals, extent of resection, or patient characteristics and comorbidities were reported.

When compared to non-irradiated, the proportion of free vascular flap success in H&N patients who received an external RT of 60–70 Gy dropped from 94 to 84 % (55). Significant vascular histology changes in the graft bed were also detected, which are dose-dependent and occur after prior (1–7 years) high-dose RT (60–70 Gy). The time interval between RT and H&N reconstructive surgery should be carefully evaluated since there is a correlation between the time elapsed after RT and the occurrence of free flap loss (53). The initial latent period before RT endothelial injuries may explain the relevance of early reconstruction following RT. In 2022, the PRADA trial demonstrated that neoadjuvant chemoradiotherapy (40–42.72 Gy in 15–16 fractions), followed by mastectomy and immediate flap reconstruction, seems safe and possible (56). At 4 weeks following surgery, 4 out of 33 (12.1%, 95% CI: 3.4–28.2) patients developed open breast wounds (>1 cm), which is comparable to post-mastectomy RT.

The majority of the studies published are based on small retrospective heterogeneous cohorts, focusing on sarcoma, H&N, and breast cancer (53, 54). Several variables may have had a role in the emergence of the contradictory results. First, patient characteristics, such as age, diabetes mellitus, previous surgery, flap type, donor vessel, timing, and postoperative anticoagulant, have been linked to poor surgical results in free tissue transfers (54). Second, the experience of the surgical team appears to be the most important factor influencing flap success (57). In fact, early complications can be prevented and managed effectively with efficient postoperative monitoring. However, one of the major significant limitations of these investigations is the inadequate evaluation of RT. Indeed, the radiation effect is caused by a dynamic interaction determined by the type of radiation, radiosensitivity, volume, location, fractionation, dose rate, and total dose of the irradiated area (58). Large randomized trials are needed to fully understand this interplay (53, 54).

The pathophysiology of delayed wound healing after RT is a dynamic process that combines cellular, extracellular, microvascular, and cytokine changes (59). Therefore, the current therapeutic options for wound healing are few, and further Level I research is required. Nonetheless, patients must be carefully evaluated and selected from the specialists participating in the multidisciplinary tumor board.

## Superficial Orthovoltage X-Rays

This technique consists of the administration of 50 or 60 kV photons directly into the area to be treated. This is used to treat superficial and small-sized skin cancers (<3 cm) that are situated on easily accessible sites (flat surfaces). Because the irradiation is so concentrated, the surrounding healthy tissues are better protected. As a result, the doses per fraction can be increased (between 5 and 20 Gy). Hence, the number of fractions decreases, limiting the patient's time in the hospital and increasing treatment compliance. Retrospective studies have shown that with definitive RT, BCC, and SCC could have a 4–5-year LC of 86–96% and 58–94%, respectively (including recurrent tumors) (60–64). Male gender, size ≥10–20 mm, lesion of the noise, and smaller fraction size have all been recognized as predictors of recurrence. These studies included a variety of RT prescriptions and techniques, such as superficial X-rays, orthovoltage, electrons, and MV photons. Contact RT can be used as an adjuvant or as an exclusive treatment. However, its utilization rate varies across the country, and it has almost disappeared in certain locations. In a retrospective registry study, Roth et al. reported that 99% of 776 NMSC (BCC: 448; SCC: 328) would not recur after 85 months following superficial RT (65). The average RT dosage was 46.53 (36.37–54.55) Gy given over 12.3 (1.85) fractions. Hypopigmentation was the most common side effect.

## Brachytherapy

For over 50 years, a number of brachytherapy (BT) techniques have been shown to be an effective treatment with a > 95% LC over 5 years, with minimal toxicity and good aesthetic outcomes (9, 66). For patients with T1-2N0M0 (AJCC or UICC 8^th^ edition), BT could be considered; techniques should be based on tumor depth of invasion, volume, and location (9, 66).

Approximately 90% of the NMSCs are less than 2 cm in diameter, and their infiltration depth is limited to a few millimeters. External beam radiotherapy, or BT, provides the best chances of recovery at this stage. Adjuvant BT is still controversial, and there is no unanimous consensus on whether it is appropriate to use it (9). However, two techniques are commonly used: contact BT (plesiotherapy) and interstitial BT (9, 66). The 2020 American Brachytherapy Society consensus generally excludes NMSC patients with an extension over subcutaneous fat, bone invasion, clinical PNI, and orbital involvement (9, 10). In all cases, the depth of infiltration of the lesion must be properly assessed (ultrasound or CT scan) to ensure sufficient dose coverage (9, 66). Contrary to popular belief, contemporary high dose rate (HDR) BT has fewer radiation protection issues than the previously employed low dose rate (LDR) BT. The much higher dosage rate requires additional shielding, but the short sessions are conducted ambulatory, eliminating the need for many days of isolation. These factors are even more important when treating elderly patients (66, 67). The single source can be utilized several times, eliminating the need for an outdated departmental “library” of LDR sources. A third, less commonly used technique is pulse dose rate (PDR) BT. The distinction between this treatment and the others is that it uses pulsed high-dose-rate irradiation, which is administered in brief “pulses” over several hours. The therapy takes one to several days and necessitates the use of a specially shielded treatment room. This approach was created to simulate the biological effect of continuous LDR BT while utilizing the benefits (i.e., full radiation shielding and the ability to optimize dose distribution) of the stepping source technology developed for HDR-BT. However, biologically it comes closer to a hyper-fractionated HDR than a continuous LDR.

SCRiBe meta-analysis compared BT (n = 553) vs. external RT in 9.965 T1-2N0 NMSC, with a median follow-up of 36 months (68). They reported less than 7% of local recurrences for both techniques at 1 year with a median dose of 45 Gy (10 fractions). Despite variations in techniques, doses, fractionations, and patient characteristics, BT gave better “good” cosmesis results than external RT (95 vs. 79 %, *p* < 0.05). In 2019, Lee et al. performed a meta-analysis comparing surgery (conventional excision or Mohs surgery) vs. external RT vs. BT, including 21.371, T1-T2N0 NMSC. BT appeared to have the best cosmetic outcomes and lowest recurrence rates over 1 year (69). However, as the cosmesis grading system was heterogenous between studies, the authors defined a consensus on the grading of each study (good vs. fair vs. poor). Moreover, only one study evaluated cosmetic outcomes in the Mohs micrographic surgery group. The rate of “excellent” or “good” cosmesis results with BT can reach up to 75 and 39%, respectively (70). The most common long-term side effects are grade 1–2 hypopigmentation, telangiectasia, and alopecia. There are two major ways to apply BT, each with its specific indication(s): contact or plesiotherapy BT and interstitial BT (9, 66).

### Contact Brachytherapy/Plesiotherapy

Contact brachytherapy can be delivered by several techniques, including surface applicators, flaps, and custom molds (Table 2).

**Table 2:** Contact brachytherapy, customized molds and flaps, Leipzig and Valencia applicators, combination of techniques.

**Author**	**Year**	**Histology**	**N. Lesions N. patients**	**Dose rate**	**N. Fractions**	**Dose/ Fraction**	**Frequency**	**Total Dose**	**Technique**	**Follow-up (median)**	**Local control**
Svoboda et al. (71)	1995	BCC (76) SCC (11) Lymphomas (2) Bowen's disease (9) Other (8)	106 lesions 76 patients	HDR	1–15	3.3–22 Gy	Daily/Twice a day/Weekly	12–50 Gy 27–50 Gy	Molds	9.6 months	100%
Allan et al. (72)	1998	BCC (9) SCC (3) Other (1)	13 patients	HDR	8	5–5.5 Gy	In five days, 1–2 times daily	42.5–45 Gy	Molds	18 months	100%
Köhler-Brock et al. (73)	1999	BCC SCC Kaposi's sarcoma Lymphomas Melanomas	520 lesions 520 patients	HDR	4–8	5–10 Gy	1–2 times a week	30–40 Gy	Surface applicators	10 years	91%
Guix et al. (74)	2000	BCC (102) SCC (34)	136 lesions 136 patients	HDR	33–36	1.8 Gy	Daily	60–65 Gy 75–80 Gy	Molds	5 years	87–98%
Skowronek et al. (75)	2005	BCC (35) SCC (20) Other (10)	179 lesions 179 patients	HDR	5–6	10 Gy	1–2 times a week	50–60 Gy	Molds/Flaps	12 months	82%
Ghaly et al. (76)	2008	BCC (35) SCC (20) Other (10)	65 lesions 59 patients	HDR	3–30	2–5 Gy	Daily/Twice a day	40 Gy	Flaps/Surface applicators	18 months	96%
Somanchi et al. (77)	2008	SCC	25 lesions 25 patients	HDR	8	5–5.6 Gy	In five days, 1–2 times a day	40–45 Gy	Molds	60 months	100%
Kanikowski et al. (78)	2009	BCC (233) SCC (118) Other (146)	497 lesions 497 patients	HDR	10 6–8	5–6 Gy 5 Gy	-	50–60 Gy 30–40 Gy	Molds/Surface applicators	12 months	72%
Maroñas et al. (79)	2011	Cutaneous carcinoma	45 lesions 32 patients	HDR	5–18	3–7 Gy	Daily/ 3 times a week	44–57 Gy	Molds/ Flaps	45 months	83–100%
Gauden et al. (80)	2013	BCC (121) SCC (115)	236 lesions 200 patients	HDR	12	3 Gy	Daily	36 Gy	Surface applicators	66 months	98%
Tormo et al. (81)	2014	BCC (45)	45 lesions 32 patients	HDR	6–7	6–7 Gy	Twice a week	42 Gy	Surface applicators	47 months	98%
Arenas et al. (82)	2015	BCC (92) SCC (42)	134 lesions 114 patients	HDR	15–19	3 Gy	3 times a week	45–57 Gy	Molds/Surface applicator	33 months	95%
Delishaj et al. (83)	2015	BCC (44) SCC (12) Kaposi's sarcoma (1)	57 lesions 39 patients	HDR	8–10	5 Gy	2–3 times a week	40–50 Gy	Surface applicators	12 months	96%
Amendola et al. (84)	2016	NMSC	42 lesions 38 patients	HDR	10	4.85–5 Gy	2 times a week	48.5–50 Gy	Molds	85 months	95%
Jumeau et al. (85)	2016	NMSC (5) Sarcoma (5) Neuroendocrine carcinoma (2)	12 lesions 11 patients	HDR	1–5	5–8 Gy	Twice a week	8–30 Gy	Molds/Flaps	17 months	91%
Kalaghchi et al. (86)	2018	BCC (45) SCC (15)	60 lesions 60 patients	HDR	10–13	3–4 Gy	Daily	30–52 Gy	Molds	24 months	88–95%
Casey et al. (87)	2019	BCC (35) SCC (20)	65 lesions 59 patients	HDR	3–30	3–5 Gy	Daily	15–60 Gy	Molds	8 months	92%
Laliscia et al. (88)	2021	BCC (23) SCC (14)	40 lesions 37 patients	HDR	8	5 Gy	2–3 times a week	25–50 Gy	Molds	25 months	90%

#### Surface Applicators

Surface applicators are shielded applicators that are enabled to treat a small (<2 cm), regular, and plane area of the skin to a maximum depth of 5 mm, using an HDR source projector. There are several types of applicators on the market, such as the Leipzig type^TM^ (Elekta or Varian) or Valencia ^TM^ (Elekta) applicator (89, 90). For each session, the patient could be immobilized using an adjustable arm, adhesive, or a thermoplastic contention. The prescribed dose depth is typically determined by the thickness of the tumor, which should be <4 mm (66). In 2013, Gauden et al. published the results of a study on 200 patients with 236 NMSCs treated with Leipzig applicators. After a median follow-up of 66 months, the local control was 98%, with good to excellent cosmetic results in 88% (80). They also reported 5.5 % of late skin hypopigmentation.

Electronic brachytherapy (EBT) has emerged as an alternative to radionuclide surface applicators for NMSCs patients (9, 66). EBT consists of an HDR X-ray source that produces radiation at relatively high-dose rates (typically 6–7 Gy at 3 Gy/min), a mobile controller that provides power and cooling to the source, and applicator sets. Axxent^TM^ (50 kV electronic source) is typically used for intracavitary treatments, whereas Intrabeam^TM^ (50 kV) and Esteya^TM^ (69.5 kV) are commonly employed for the skin.

Like radionuclide HDR-BT, the miniature X-ray source can be used along the catheter to individualize/optimize treatment. Accelerating electrons strike a tungsten target, producing low-energy photons. Because of their extremely low energy, protected rooms are no longer required. The clinical implementation, dosimetry, and output verification of skin applicators have all been thoroughly documented (91). In terms of long-term outcomes and comparison to conventional treatment, the data for electronic brachytherapy is currently insufficient (9). However, based on a short follow-up, the results appear to be highly promising in terms of local control and toxicity (Table 3) (9). After a median follow-up of 10 months, the treatment of 171 NMSC lesions with EBT (40 Gy in 8 fractions, twice weekly) showed no relapse and good (93%) cosmesis (92). A prospective single non-randomized study on 40 nodular and superficial BCC patients treated with six fractions of 6.1 or 7 Gy demonstrated a 1-year LC of 90 and 95%, respectively. This study shows that the conventional prescription had a higher LC without a significant difference in acute, late, or cosmesis outcomes (96). The American Brachytherapy Society currently recommends against using EB outside of a prospective clinical registry or trial (100).

**Table 3:** Surface applicators, electronic brachytherapy.

**Author**	**Year**	**Histology**	**N. Lesions N. patients**	**N. Fractions**	**Dose/Fraction**	**Total dose**	**Frequency**	**Median follow up**	**Local control**
Bhatnagar et al. (92)	2013	NMSC	171 lesions 122 patients	8	5 Gy	40 Gy	Twice a week	10 months	100%
Doggett et al. (93)	2015	NMSC	524 lesions 524 patients	8	5 Gy	40 Gy	Twice a week	12.5 months	100%
Paravati et al. (94)	2015	BCC (149) Other (5)	154 lesions 127 patients	8	5 Gy	40 Gy	Twice a week	16.1 months	99%
Goyal et al. (95)	2015	BCC (20) SCC (3)	23 lesions 19 patients	8–20 (mean 10)	4 Gy – in 20 lesions	Mean 40 (32–50)	Daily	12 months	100%
Ballester-Sanchez et al. (96)	2016	BCC (40)	40 lesions 40 patients	6 6	6.1 Gy 7 Gy	36.6 Gy 42 Gy	Twice a week	At least 1 year	90% 95%
Bhatnagar et al. (97)	2016	BCC (990) SCC (670) Other (88)	1,822 lesions 1,259 patients	3–8	5 Gy	40–45 Gy	2–3 times a week	4–16 months	99%
Patel et al. (98)	2017	BCC (113) SCC (95)	208 lesions 188 patients	8–10	4–5 Gy	32–50 Gy	Twice a week	3.3 years	99.5%
Goyal et al. (95)	2021	BCC (28) SCC (22)	50 lesions 33 patients	8	5 Gy	40 Gy	Twice a week	45.6 months	98%
Cerrolaza et al. (99)	2021	BCC (19) SCC (5) Other (11)	35 lesions 35 patients	8–10	5 Gy	40–50 Gy	1–2 times a week	4.6 months	100%

#### Surface Flaps

Flaps make it possible to treat deeper lesions (≤ 5 mm) on a uniform or slightly curved surface. They consist of catheters (guides with a diameter of 1.6 to 2 mm, 5 or 6 French) wherein the radioactive source circulates stepwise. The different catheters are interconnected and form a moderately flexible flat surface. This device is attached to the skin; no anesthesia is required. A minimum of 5 mm to the skin and a gap of 10 mm between catheters are required, and the typical prescription depth is <5 mm under the skin (66). However, hand-made flaps of different sizes and with varying gaps between sources can be made by the practitioner to meet the needs of the individual surface. The standard prescription depth is <5 mm under the skin (66). Nonetheless, several models have already been made with catheters placed at an exact distance from the surface to be treated, such as the Freiburg^TM^ Elekta Flap, the Catheter Flap Set^TM^ from Varian, and the HAM^TM^ by Mick Radio Nuclear Instrument (66). Flaps can be cleaned, sterilized, and reused.

#### Custom MOLDS

In the case where irregular anatomical areas are to be irradiated, such as the nose and the outer ear, custom molds can be made by individual prints from polymers/acrylic, resin, wax, or a thermoplastic regulatory approval material (66). Currently, some centers use 3D printers to make a mold that perfectly matches the surface that has be treated (101). Kalaghchi et al. reported a complete clinical response rate of 95% in NMSC (median size = 3 cm) H&N patients at 3 months following HDR-BT using alginate custom molds (86). In the adjuvant and definitive BT groups, the recurrence rate was around 11 and 2%, respectively. Furthermore, 96% of patients showed good/excellent cosmetic outcomes after 2 years. Maroñas et al. used custom molds to treat 51 carcinomas of the facial skin (mean size = 1.5 cm). Five lesions relapsed, and four were located at the tip of the nose because the applicator did not completely cover the margins. It is, therefore, essential to adapt the procedures according to the locations (79). Molds must fit the patient's surface, and catheters must remain near the tumor surface to obtain optimal dosage coverage. A thermoplastic mask with catheters could be useful for managing the intra and inter-fractional motion. The prescription point is typically 3–5 mm under the skin's surface. For flaps/molds, dose and fractionation regimens include 35–50Gy, 10–20 fractions or 60–70, 30–35 fractions (9).

### Interstitial Brachytherapy

Contact techniques can treat lesions up to 5 mm deep; above this depth, interstitial implants are required. Currently, interstitial LDR BT gradually left room for HDR-BT (4, 66, 70). This approach requires local or (loco-)regional anesthesia, which is recommended, as it minimizes volume fluctuations when the local anesthetic is injected for catheter placement (not necessary for removal). CT-scan images (≤ 2 mm) are recommended for catheter and target region reconstruction (66). Generally, one level of catheters is sufficient for NMSC cancers, but deep/thick lesions (>10 mm) may require several levels/layers. Optimisation software in HDR can compensate for minor differences in catheter distance (66). Some space and catheter stabilization techniques are useful for maintaining the geometry of multi-catheter interstitial implants (76). Plastic tubes are more malleable and are the first choice for non-planar surfaces, whereas metallic needles give better stability (66). To avoid telangiectasia, necrosis, or delayed healing, the catheters should be placed 3–4 mm under the skin surface (66). Usually, the patient is treated twice a day, at least 6 hours apart. In HDR, a high dose per fraction is used, with the total dose ranging from 30–55 Gy in 8–10 fractions (60–70 Gy in a continuous flow of 0.75 Gy/h in PDR). Fractionation and dose depend on location and the type of treatment (exclusive or adjuvant); hence no clear recommendation can be made because of the wide variety of published doses, more based on experience than on evidence (9, 66). Most data are based on the good results of studies published in LDR. Rio et al. treated 97 patients with peri-orificial facial skin carcinomas. The local control rate was 92.5% for cases treated with radical intent and 88% for postoperative BT (median FU of 55 months) (102). Most treatment planning systems for BT calculation rely on the American Association of Physics in Medicine (AAPM) 43 report (103).

## Conclusion

Radiation therapy plays a major role in the management of NMSCs. Whether exclusive or adjuvant, it has shown very good results in terms of local control and cosmetic results. New techniques such as contact brachytherapy and electronic brachytherapy are promising new treatments. However, good, randomized trials explaining the variation in recommendations on dose and fractionation are lacking. New systemic therapies will be developed in the near future. We will have to define their roles and take into account all these modalities to offer the best care to our patients. Due to the excellent results of radiotherapy and HFRT for the elderly, a multidisciplinary consultation, including a radiation-oncologist, is essential to provide the most effective care.

## Author Contributions

Study design: AD and SB. Data collection: SB, AD, and CG. Data analysis, interpretation, and writing of the manuscript: AD, SB, CG, and DV. Revision of the manuscript: SB, DV, CG, SP, and AD. All authors contributed to the article and approved the submitted version.

## Conflict of Interest

The authors declare that the research was conducted in the absence of any commercial or financial relationships that could be construed as a potential conflict of interest.

## Publisher's Note

All claims expressed in this article are solely those of the authors and do not necessarily represent those of their affiliated organizations, or those of the publisher, the editors and the reviewers. Any product that may be evaluated in this article, or claim that may be made by its manufacturer, is not guaranteed or endorsed by the publisher.
